# Silica-Encapsulated Perovskite Nanocrystals for X-ray-Activated Singlet Oxygen Production and Radiotherapy Application

**DOI:** 10.1021/acsenergylett.3c00234

**Published:** 2023-03-17

**Authors:** Francesco Carulli, Mengda He, Francesca Cova, Andrea Erroi, Liang Li, Sergio Brovelli

**Affiliations:** ⊥ Università degli Studi di Milano-Bicocca, Dipartimento di Scienza dei Materiali, Via Cozzi 55, 20125 Milan, Italy; ‡ School of Environmental Science and Engineering, Shanghai Jiao Tong University, Shanghai 200240, China; § Macao Institute of Materials Science and Engineering (MIMSE), Macau University of Science and Technology, Taipa 999078, Macao, China

## Abstract

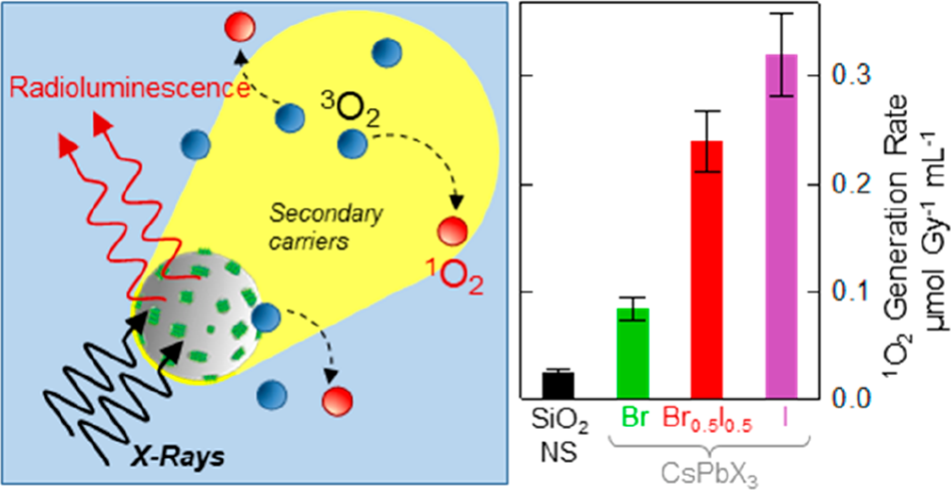

Multicomponent systems consisting of lead halide perovskite nanocrystals (CsPbX_3_-NCs, X = Br, I) grown inside mesoporous silica nanospheres (NSs) with selectively sealed pores combine intense scintillation and strong interaction with ionizing radiation of CsPbX_3_ NCs with the chemical robustness in aqueous environment of silica particles, offering potentially promising candidates for enhanced radiotherapy and radio-imaging strategies. We demonstrate that CsPbX_3_ NCs boost the generation of singlet oxygen species (^1^O_2_) in water under X-ray irradiation and that the encapsulation into sealed SiO_2_ NSs guarantees perfect preservation of the inner NCs after prolonged storage in harsh conditions. We find that the ^1^O_2_ production is triggered by the electromagnetic shower released by the CsPbX_3_ NCs with a striking correlation with the halide composition (I_3_ > I_3–*x*_Br_*x*_ > Br_3_). This opens the possibility of designing multifunctional radio-sensitizers able to reduce the local delivered dose and the undesired collateral effects in the surrounding healthy tissues by improving a localized cytotoxic effect of therapeutic treatments and concomitantly enabling optical diagnostics by radio imaging.

In the last decades, interest in nanoparticles in the biomedical field experienced a rapid growth due to the tunability of their physical and chemical properties and their rich surface chemistry that enables specific functionalization by design.^1,2^ Different classes of functional nanoparticles, including metals, semiconductors,^3,4^ metal/lanthanide oxides,^5,6^ and organic or hybrid systems,^7,8^ have found successful application in several medical branches, such as nanotherapy, diagnostics, and imaging.^9−11^ Today, one of the most advanced biomedical uses of nanoparticles is offered by their strong interaction with ionizing radiation, which makes it possible to improve the effectiveness of conventional cancer treatments^12^ and imaging techniques.^13^ In oncological therapies, one of the most adopted medical treatments is radiotherapy (RT, ca. 50% of total cases),^14,15^ a noninvasive technique typically consisting of the local release of the energy of X-rays via photoelectric effect and/or Compton scattering to stop tumor cell proliferation, either directly by damaging their DNA or indirectly by forming cytotoxic free radicals—such as singlet oxygen (^1^O_2_), superoxide (O^2–^) or hydrogen peroxide (H_2_O_2_)—commonly termed reactive oxygen species (ROS), upon interaction with the cellular aqueous environment.^16^ Currently, in order to achieve significant therapeutic effects, patients are exposed to high doses of X-ray radiation (typically 40–60 Gy in a complete RT treatment) that carry a high risk of damaging surrounding healthy areas, due to the difficulty of finely focusing the radiotherapy exclusively on the region of interest.^17^ In order to reduce X-ray exposure, several strategies have been proposed to increase the local ROS production, such as radio-stimulated photodynamic therapy and radiation catalysis.^2,18^ The first is based on activating a photosensitizer responsible for the energy transfer to O_2_ molecules promoting ROS production, whereas the second takes advantage of the chemical and catalytic activities of nanoparticle surfaces to enhance the generation of radiation-induced radicals by, for example, water radiolysis.^19^

Metal halide nanocrystals (NCs),^20−25^ both in their most common lead-based inorganic or hybrid perovskite form (APbX_3_, with A = Cs, methylammonium, formamidinium, X = Cl, Br, I)^26−28^ or in lead-free alternatives,^29−31^ have recently attracted substantial attention for ionizing radiation detection,^32^ prized for their high average atomic number (*Z*) that enhances the interaction probability with ionizing radiation (*P*_i_ ∼ *Z*^*n*^, with *n* = 1–5 depending on the type of radiation and interaction),^33^ efficient scintillation,^34−36^ and strong robustness to prolonged exposure to ionizing radiation.^35^ Importantly, the easy tuning of their emission spectrum from UV to NIR further makes them interesting candidates as biological markers for radio-imaging, naturally overcoming the limitations of common fluorophores to fit the near-infrared transparency window of biological tissues.^37,38^ This opens up perspectives for the simultaneous application of LHP NCs in diagnostics and therapeutics, acting as X-ray biological markers to identify and target diseased areas and simultaneously as sensitizers for enhanced radiotherapy.^39^

Despite such promise, very few examples of medical diagnostic and therapeutic strategies based on metal halide NCs have been proposed,^40−42^ mostly because of their low stability in aqueous environment^43^ resulting in their rapid dissolution and further consequent release of potentially harmful Pb^2+^ ions. Recently, innovative strategies for the realization of high-quality CsPbX_3_ NCs inside impermeable host matrixes have been proposed,^44−53^ including mesoporous SiO_2_ particles,^54−61^ semiconducting shells,^62,63^ metal–organic frameworks,^64^ glasses and metal oxides,^65−71^ which preserve the luminescence properties of the host NCs even in harsh environments and prevent Pb dispersion in the surroundings,^29,72,73^ effectively removing the constraints for the application of this class of materials in biological environments. To date, however, no study has approached the use of metal halide NCs for radiotherapy.

In this work, we aim to contribute to this endeavor by demonstrating that CsPbX_3_ (X = Br, I) NCs directly synthesized inside mesoporous silica nanospheres (SiO_2_–NSs) behave as effective X-ray sensitizers for the generation of ^1^O_2_ species, boosting the effect of bare SiO_2_ NSs by over 10-fold. Interestingly, we found that the ^1^O_2_ sensitization effect is largely due to the release of secondary electrons by the CsPbX_3_–SiO_2_ NSs without quenching their radioluminescence (RL) and that neither the RL nor the photoluminescence (PL) are affected by high radiation doses or by prolonged storage in an aqueous environment (even in highly acid solutions). These results, combined with the inhibition of Pb^2+^ cation leakage outside the NS, made possible by the perfect sealing of the pores, open up the future possibility of implementing CsPbX_3_–SiO_2_ NSs as radio-stimulated markers and therapeutic agents.

CsPbX_3_–SiO_2_ NSs of different halide composition (namely, CsPbBr_3_, CsPbBr_1.5_I_1.5_, and CsPbI_3_) were synthesized using SiO_2_ NS as templates by a solid-state confined growth technique in the presence of potassium salt as sintering agent, which promotes complete collapse of the porous structure, isolating the inner CsPbX_3_ NCs from the outer environment and maintaining good solubility of the NSs in water. Specifically, spherical SiO_2_ NSs with diameter ∼200 nm and even distribution of internal pore dimensions were dispersed in a distilled water solution containing a proper proportion of the NC precursors (see Methods in the Supporting Information for details) and kept under stirring to favor the soaking of ions inside the pores. CsPbX_3_ NCs were subsequently synthesized inside the pores by drying at 80 °C to remove excess solvent followed by heating at 600 °C in the presence of potassium salt (K_2_CO_3_ and KI, respectively) to trigger the calcination reaction (details of the effects of the calcination temperature and conditions are reported in ref (54)). Besides prompting the formation of CsPbX_3_ NCs, the high temperature also favors the full collapse of the SiO_2_ pores, which encloses the NCs inside the NSs and protects them from oxidation and ripening fusion. After cooling to room temperature, the CsPbX_3_–SiO_2_ NSs were washed with ultrapure water several times to remove unreacted precursors and possible products formed outside the NSs, collected via centrifugation, dried at 60 °C, and finally redispersed in water for further studies.

Transmission electron microscopy (TEM) images of CsPbX_3_–SiO_2_ NSs are reported in Figure 1a and show spherical nanoparticles comparable to the original template NSs (see Figure S1) with a slight reduction in size due to pore collapse and subsequent shrinkage during calcination (see Figure S2), without any aggregation due to interparticle cross-linking. High-angle annular dark-field scanning transmission electron microscopy (HAADF-STEM) images and the corresponding elemental mappings (Figure 1b) of CsPbBr_3_, CsPbBr_1.5_I_1.5_, and CsPbI_3_ show that all constituent elements (Cs, Pb, Br, and I) are detected only at the NS structure, indicating that no NC remained outside the particles. Also importantly, the adopted calcination procedure maintains high particle solubility in aqueous solvent, which is a fundamental aspect to allow their applicability (as shown in Figure 1c). The crystal structure of the as-synthesized CsPbX_3_ NCs inside the SiO_2_ NSs and respective size distributions were studied via X-ray diffraction (XRD) and TEM, as reported in Figures 1d and S3, respectively. The XRD patterns show, in every case, a broad diffraction peak at 23° due to the contribution of amorphous SiO_2_. The diffraction peaks of the CsPbBr_3_–SiO_2_ NSs at 21.36°, 26.32°, and 30.42° match cubic CsPbBr_3_ structure. Consistent with their mixed halide composition, the XRD pattern of CsPbBr_1.5_I_1.5_–SiO_2_ NSs shows the coexistence of cubic CsPbBr_3_, the emissive γ-phase of CsPbI_3_ (peaks at 20.09°, 28.48°, and 28.92°) together with traces of the optically passive orthorhombic CsPbBr_1.5_I_1.5_ δ-phase (peaks at 27.21°, 25.70°, and 31.31°); this is expected considering the thermodynamically favored crystalline transition of the CsPbI_3_ γ-phase into the δ-phase below 150 °C.^74^ Finally, the spectrum of CsPbI_3_–SiO_2_ NSs shows the γ-phase peaks and a more prominent contribution by the δ-phase. The emission properties of the CsPbX_3_–SiO_2_ NSs were studied using optical and X-ray excitation, and the corresponding PL and RL spectra are reported in Figure 1e. Consistent with previous results, both the PL and RL spectra progressively shift from the green to the NIR spectral regions with increasing iodine content.^21^ In all three samples, the PL spectra show the narrow peak due to excitonic emission, indicating the absence of side products or emitting defect states introduced by the confined growth in the SiO_2_ NS templates. The PL quantum efficiency was found to be 55 ± 5%, 21 ± 4%, and 12 ± 3% for CsPbBr_3_, CsPbBr_1.5_I_1.5_, and CsPbI_3_, respectively.

**Figure 1:**
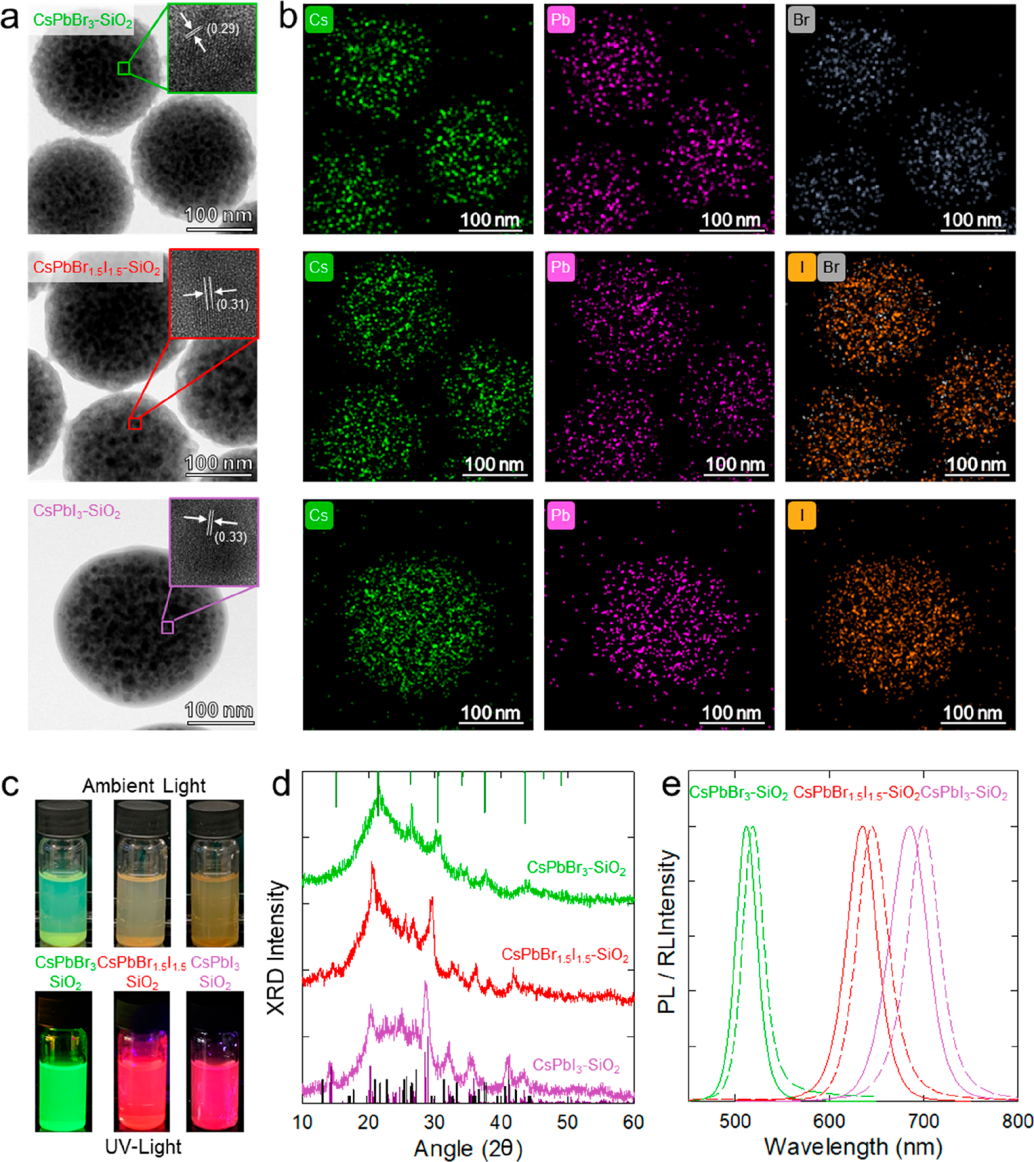
(a) TEM images of CsPbBr_3_–SiO_2_, CsPbBr_1.5_I_1.5_–SiO_2_, and CsPbI_3_–SiO_2_ NSs. (b) HAADF-STEM images and elemental mappings on the same samples highlighting the presence of Cs (green), Pb (purple), Br (gray), and I (yellow) inside the NSs. (c) Photographs of CsPbBr_3_–SiO_2_, CsPbBr_1.5_I_1.5_–SiO_2_, and CsPbI_3_–SiO_2_ in aqueous solution taken under ambient illumination (top pictures) and under UV illumination (bottom pictures). (d) XRD patterns of CsPbBr_3_–SiO_2_ (green line), CsPbBr_1.5_I_1.5_–SiO_2_ (red line), and CsPbI_3_–SiO_2_ (purple line). The diffraction patterns of cubic CsPbBr_3_ (ICSD 97852, green), orthorhombic γ-phase (ICSD 434338, violet line), and orthorhombic δ-phase (ICSD 250744, black line) of CsPbI_3_ are also reported as references. (e) Normalized PL (solid lines) and RL (dashed lines) spectra of the same samples in dry powder form (excitation wavelength, 405 nm for PL; X-ray irradiation at 20 kV for RL).

The PL decay time of all NSs reported in Figure S4 is consistent with previous reports and features a dominant radiative fast component followed by a minor contribution due to delayed fluorescence by back-transfer from shallow traps.^35^ The RL spectra are slightly red-shifted compared to the respective PL, which possibly originates from the radiative recombination of shallow emissive defect states in the proximity of energy bands typically due to halide surface vacancies as already observed in colloidal CsPbBr_3_ NCs.^35,75^

Next, we proceeded with validating the potential of CsPbX_3_–SiO_2_ NSs as X-ray sensitizer by studying the production of the singlet oxygen (^1^O_2_) species in aqueous environment under X-ray irradiation. In these experiments, schematically depicted in Figure 2a, we dispersed identical concentrations of CsPbX_3_–SiO_2_ NSs (2 mg/mL) with different halide composition in a phosphate buffer solution (PBS) to artificially mimic the physiological pH conditions; the same experiment was performed with bare SiO_2_ NS as reference. The commercially available fluorescent probe singlet oxygen sensor green (SOSG) was used to monitor *in situ* the ^1^O_2_ evolution. In its unoxidized form, SOSG is nonemissive, whereas its endoperoxide derivative formed upon oxidation by ^1^O_2_ exhibits a characteristic PL band at 530 nm (Figure S5). Therefore, the SOSG PL intensity can be used to quantify the ^1^O_2_ concentration during X-ray irradiation. The PL spectra of SOSG excited at 473 nm with a cw laser in PBS solutions of CsPbX_3_–SiO_2_ NSs were collected during 10 min of continuous exposure to soft X-rays (*E*_max_ = 20 keV) with 0.5 Gy/s dose rate. The X-ray excitation of CsPbX_3_–SiO_2_ NSs simultaneously triggers the sensitization of ^1^O_2_ production and the NSs RL. In fact, the ability to emit RL while simultaneous sensitizing ROS production represents an important feature of our systems with respect to common radio-activated photosensitizers such as porphyrin-based assemblies that produce ^1^O_2_ via energy transfer to the triplet states of molecular O_2_ at the expense of their luminescence. Such a feature, combined with preserving their RL emission after significant amount of radiation (as shown in Figure 2b), offers the possibility of using CsPbX_3_–SiO_2_ NSs also as efficient radio-stimulated bio markers for *in vitro* or *in vivo* radio-imaging.

**Figure 2:**
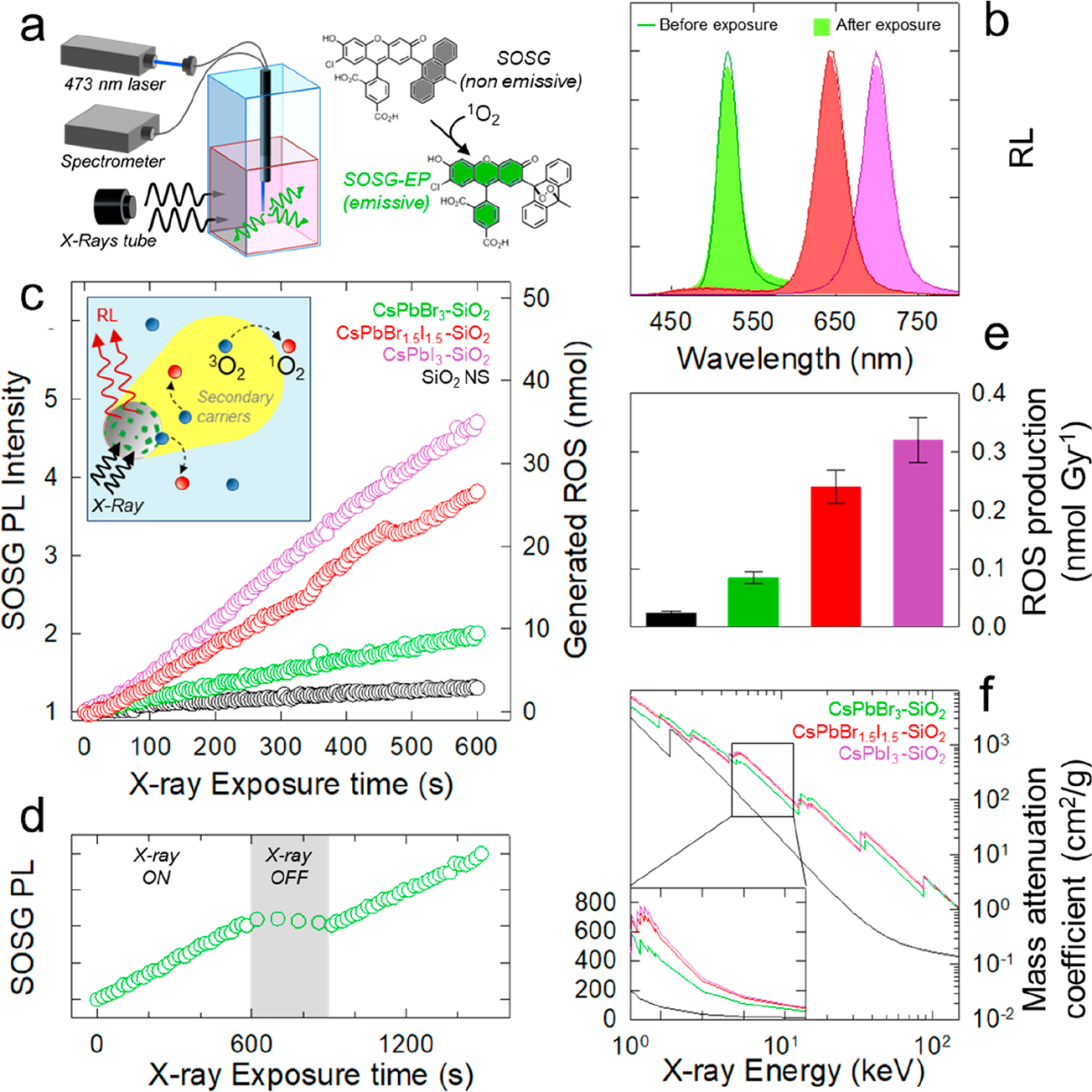
(a) Sketch of the experimental setup for the measurement of ^1^O_2_ production under X-ray irradiation. (b) RL spectra of CsPbBr_3_–SiO_2_, CsPbBr_1.5_I_1.5_–SiO_2_, and CsPbI_3_–SiO_2_ NSs before (lines) and after (shaded areas) exposure to 20 Gy dose of X-rays. The same color code is applied to all panels. (c) SOSG PL intensity (excited at 473 nm) normalized for the initial value for pristine SiO_2_ NS (black circles), CsPbBr_3_–SiO_2_ (green circles), CsPbBr_1.5_I_1.5_–SiO_2_ (red circles), and CsPbI_3_–SiO_2_ (purple circles) as a function of X-ray exposure time. Inset: Sketch of ROS production mechanism. (d) SOSG PL intensity excited at 473 nm during the full X-ray irradiation sequence in the presence (X-ray ON) and in the absence of simultaneous X-ray irradiation (X-ray OFF) for a solution containing CsPBI_3_–SiO_2_ NSs. (e) ^1^O_2_ production rate calculated from the linear fitting of the data in panel c. (f) X-ray mass attenuation coefficient of the investigated material systems based on the NIST database.^76^ In the inset the enlargement of the mass attenuation coefficient in linear scale in the energy range of the X-rays used in our experiments is reported.

In Figure 2c we report the integrated intensity of the SOSG PL during the scan for the CsPbX_3_–SiO_2_ NSs as well as for the control solution containing bare SiO_2_ NS; on the right axis we report the respective ^1^O_2_ concentrations as extracted via the calibration procedure described in the Supporting Information. Notably, all solutions containing CsPbX_3_–SiO_2_ NSs exhibit systematically higher ^1^O_2_ production with respect to bare SiO_2_ NSs, with a 3-fold, 10-fold, and 13-fold enhancement along the series CsPbBr_3_–SiO_2_, CsPbBr_1.5_I_1.5_–SiO_2_, and CsPbI_3_–SiO_2_ NSs, which indicates the substantial effect of the CsPbX_3_ NCs on the ^1^O_2_ generation.

Importantly, as shown in Figure 2d for the CsPbI_3_–SiO_2_ NSs (the other samples are reported in Figure S7), when the X-ray irradiation was momentarily interrupted and the solution was excited solely by the 473 nm laser, no additional ^1^O_2_ was created, and the trend proceeded identically only after the X-irradiation was reestablished. This is relevant since the PL of CsPbI_3_–SiO_2_ NSs at 685 nm (1.81 eV) excited by the 473 nm laser source is partially resonant to the triplet state of O_2_ (1.62 eV^77^) and could, in principle, produce ^1^O_2_ via nonradiative energy transfer, similar to what occurs with common radio-activated photosensitizers.^78,79^ The absence of ^1^O_2_ production without X-rays therefore indicates that the process is a direct result of the interaction of ionizing radiation with the CsPbI_3_–SiO_2_ NSs with negligible mediation by its excitonic states; we note that the absence of ET despite the energy resonance could be due to the relatively large distance between the NCs and the particle surfaces imposed by the calcination procedure, as well as by the relatively fast decay time of the NC PL (4–8 ns) with respect to the micro-to-millisecond PL of sensitizer phosphorescence.^79,80^ Consistent with the ^1^O_2_ production being dominated by the interaction probability between the CsPbX_3_–SiO_2_ NSs and the X-rays, we found remarkably good correlation between the ^1^O_2_ production rate and the halide composition. Specifically, as shown in Figure 2e, in which we report the ^1^O_2_ production rate extracted from the linear fitting of the curves in Figure 2c, the NSs containing iodine-based NCs, namely CsPbBr_1.5_I_1.5_–SiO_2_ and CsPbI_3_–SiO_2_, exhibited a substantially higher ^1^O_2_ generation rate compared to the CsPbBr_3_–SiO_2_. This trend correlates well with the mass attenuation coefficient of the systems reported in Figure 2f (calculated using the NIST database^76^ and EDX analysis reported in Figure S8) in the energy range of the soft X-rays used in our experiments, which is a direct consequence of their halide composition (with the other constituents being identical). As expected based on the higher *Z* of I with respect to Br (53 vs 35), the mass attenuation coefficient of the NCs monotonically grows with increasing iodine content, which results in increasing release of energy in the surrounding environment and subsequently larger ^1^O_2_ generation rate. In fact, in the case of X-rays, the primary interactions occur by photoelectric effect or by inelastic Compton scattering, resulting in an avalanche of highly energetic secondary carriers that release their energy while traveling through a medium resulting in its ionization/excitation. Since the free path of secondary carriers is typically longer than the NS size, a substantial fraction of energy effectively escapes from the nanoparticle and is released for long distances along the ionization track, leading, in our case, to the observed strong sensitization of ^1^O_2_ production.^19^

Notably, recent studies^81,82^ demonstrated that a significant fraction of energy is deposited within the nanoparticles despite the primary interaction being shared between the nanoparticles themselves and the surrounding aqueous media. Indeed, energetic secondary charges exhibit migration ranges in most cases larger than the small nanoparticle size; consequently, a fraction of energy escapes from the nanoparticle and is released for long distances along the ionization track, directly activating the ROS production in water and the direct DNA cell damage.

Based on the promising ^1^O_2_ sensitization rate of CsPbI_3_–SiO_2_ NSs, we further assessed their radiation resistance after 60 Gy, corresponding to the total dose that an RT patient cumulates in the entire RT treatment, as reported in Figure S9, in which the RL spectra collected before and after irradiation show that CsPbI_3_–SiO_2_ retained more than 80% of its initial RL emission intensity.

Finally, to further corroborate the potential suitability of CsPbX_3_–SiO_2_ NSs for X-ray stimulated applications, we assessed the risk related to the potential contamination of the environment by leakage of Pb atoms.

For this purpose, we monitored the concentration of Pb^2+^ in a water solution containing CsPbX_3_–SiO_2_ NSs (0.5 mg/mL) for 42 days by means of inductively coupled plasma–optical emission spectrometry (ICP-OES). The results, reported in Figure 3a, highlight that the concentration of Pb^2+^ was close to the sensitivity of ICP-OES, which settles a detection limit of 10 μg/L for the Pb^2+^ concentration. Considering the initial concentration of our CsPbX_3_–SiO_2_ NSs, we estimate that the amount of released Pb^2+^ in the monitored period is well below the 5 μg/g threshold established by the World Health Organization. To offer an illustrative comparison, if we consider a radiotherapy treatment involving the use of 10 mg of CsPbX_3_–SiO_2_ for a period of time comparable to our test, the total amount of lead introduced into the body would be equal to that which would be obtained by consuming 250 g of white rice.

**Figure 3:**
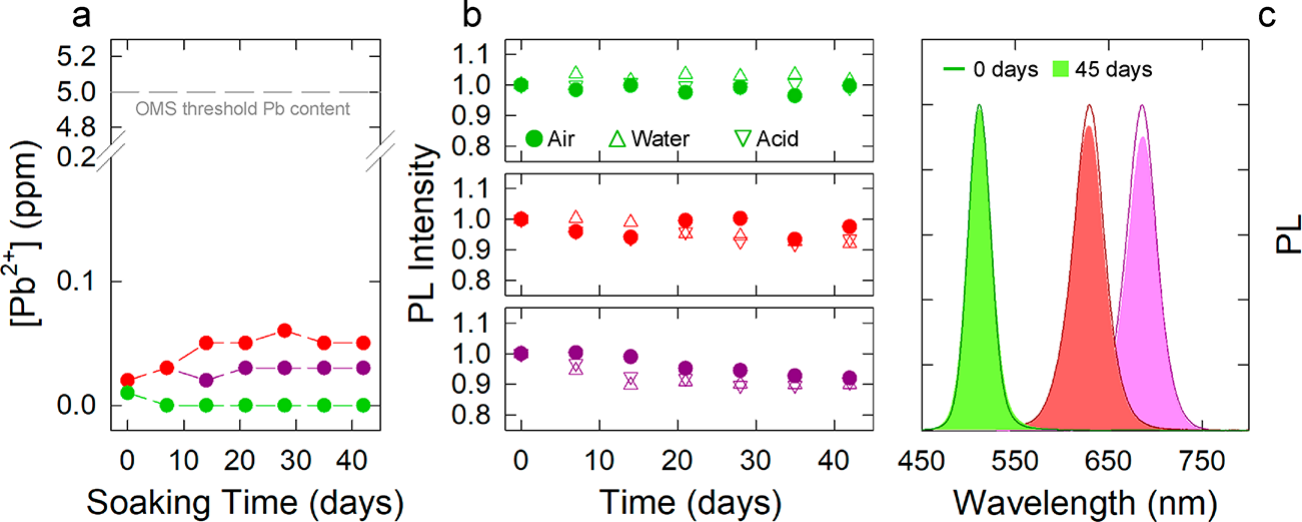
(a) Residual concentration of Pb^2+^ in 2 mg/mL solution of CsPbBr_3_–MSN (green plot), CsPbBr_1.5_I_1.5_–MSN (red plot), and CsPbI_3_–MSN (violet plot) in water as a function of soaking time. The concentrations were measured through inductively coupled plasma–optical emission spectrometry (ICP-OES). (b) PL intensity of CsPbBr_3_–MSN (green markers), CsPbBr_1.5_I_1.5_–MSN (red markers), and CsPbI_3_–MSN (violet markers) as a function of time in different storage conditions: in air (filled circles), in water solution (triangles up), in strong acid solution (HCl 1M, triangles down). PL emission was excited with a 405 nm laser. (c) Representative PL spectra of CsPbBr_3_–MSN (green plot), CsPbBr_1.5_I_1.5_–MSN (red plot), and CsPbI_3_–MSN (violet plot) at different soaking time in acid solution (pH 1) showing no modification of emission profile.

Finally, we monitored the optical properties of our CsPbX_3_–SiO_2_ NSs in ambient atmosphere (55% humidity), water, and acid solution (1 M HCl, pH 1) in order to assess their long-time stability in conditions of potential biological interest. As shown in Figure 3b,c, nearly identical trends are observed in any condition, with nearly complete retention of the PL intensity for the CsPbBr_3_–SiO_2_ and CsPbBr_1.5_I_1.5_–SiO_2_ NSs and a slight (ca. 10%) loss for the CsPbI_3_–SiO_2_ NSs, and the spectral properties are perfectly retained by all systems (complete spectra are reported in Figure S10).

In summary, we synthesized and studied ultrastable CsPbX_3_–SiO_2_ NSs combining the strong interaction probability and scintillation features of lead halide NCs with the robustness of silica. We demonstrated that such CsPbX_3_–SiO_2_ NSs dramatically sensitize the production of ^1^O_2_ in water under X-ray stimulation and that the generation rate correlates well with their halide composition that leads to marked differences in their mass attenuation coefficient. Our data further indicate that the ^1^O_2_ production is a direct result of the release of highly energetic secondary carriers in the environment and does not require quenching of their radioluminescence, thus potentially enabling their use as both therapeutic agents and radio-markers. Finally, we proved that our NSs retain their optical properties in aqueous and harsh pH conditions and under prolonged exposure to ionizing radiation. These results offer guidelines for the design of high-*Z* radio-sensitizers for enhancing the localized therapeutic effect of RT, reducing the delivered dose and consequent damage toward healthy tissues and thus potentially improving the quality of life of patients during and after radiological treatments.

## Supporting Information Available

Detailed description of the NS synthesis and experimental methods; characterization data such as MSN and NC size distribution, time-resolved PL, temperature, and stability of PL spectra in acidic and basic solutions (PDF)
