# Supplementary material for: Silica-Encapsulated Perovskite Nanocrystals for X-ray-Activated Singlet Oxygen Production and Radiotherapy Application
Source: ACS Energy Lett. 2023 Mar 17;8(4):1795–802. doi: 10.1021/acsenergylett.3c00234 (PMC10111416; doi:10.1021/acsenergylett.3c00234)
Supplement: Supplementary file 1 — nz3c00234_si_001.pdf [file nz3c00234_si_001.pdf]

Supplementary Information

Silica-encapsulated perovskite nanocrystals for X-ray-activated singlet oxygen production and radiotherapy applications

Francesco Carulli[1†], Mengda He[2†], Francesca Cova[1]†, Andrea Erroi[1], Liang Li[4*] and Sergio Brovelli[1*]*

[1] *Università degli Studi di Milano-Bicocca, Dipartimento di Scienza dei Materiali, Via Cozzi 55, 20125 Milan, Italy*
[2] *School of Environmental Science and Engineering, Shanghai Jiao Tong University, Shanghai 200240, China*
[3] *Macao Institute of Materials Science and Engineering (MIMSE), Macau University of Science and Technology, Taipa 999078, Macao, China*

† These authors contributed equally to this work

Methods

Chemicals. Cesium bromide (CsBr, 99.5%), cesium iodide (CsI, 99.9%), lead bromide ($PbBr_2$, 99%), lead iodide (PbI_2 99%) Potassium carbonate (K_2CO_3, 99%), cetyltrimethylammonium bromide (CTAB, 95%) and tetraethylorthosilicate (TEOS, 95%), sodium hydroxide (NaOH, 98%), cesium carbonate (Cs_2CO_3, 99.9%), 1-octadecene (ODE, 90%), oleylamine (OAm, 90%) were purchased from Aladdin. Oleic acid (OA, 90%) was purchased from Aldrich. F127 ($EO_{106}PO_{60}EO_{106}$, AR) was purchased from Macklin. Ethanol (99.5%), Methyl acetate (98%) and toluene (99.5%) were purchased from Sinopharm Chemical Reagent. meso-tetra(4-sulfonatophenyl) porphyrin (H_2TPPS^{4-}) was purchased from Combi-Blocks. All the chemicals were used without further purification.

$CsPbX_3$-SiO_2 synthesis. The preparation of LHP was performed following a previously optimized procedure. Specifically, 0.6 mmol of salt precursors (127.69 mg of CsBr and 220.20 mg of $PbBr_2$ for $CsPbBr_3$, 63.8 mg of CsBr, 77.9 mg of CsI, 110.1 mg of $PbBr_2$ and 138.3 mg of PbI_2 for $CsPbBr_{1.5}I_{1.5}$, 155.9 mg of CsI and 276.6 mg of PbI_2 for $CsPbI_3$) were dissolved in 50 mL ultrapure water, sonicated for 5 min and stir continuously at 80 °C for 30 min until clear. Then around 1050 mg of MSNs pre-dispersed in 20 mL ultrapure water (the mass ratio of LHP precursors: MSNs=1:3) was added into the above solution. After stirring for 20min, the potassium salt was added (the mole ratio of $CsPbX_3$: K = 1:1). The mixture was stirred continuously at 80°C until dry. The collected mixture was ground and calcined at 600°C for 30min with a heating rate of 5 °C/min in a muffle furnace under air atmosphere for $CsPbBr_3$ and Ar atmosphere for $CsPbBr_{1.5}I_{1.5}$ and $CsPbI_3$. After cooling to room temperature, the sample was ground and washed with ultrapure water several times to remove external LHP or other salts. Finally, the washed sample was obtained by centrifugation and drying at 80 °C. The resultant composites were denoted as $CsPbBr_3$-SiO_2, $CsPbBr_{1.5}I_{1.5}$- SiO_2 and $CsPbI_3$- SiO_2, respectively.

Morphological and elementary characterization. The powder X-ray diffraction (XRD) patterns of samples were performed by a Bruker D8 Advance X-ray Diffractometer at 40 kV and 30 mA using Cu Kα radiation (λ = 1.5406 Å). The morphologies and elemental distributions and high-angle annular dark field scanning

transmission electron microscopy (HAADF STEM) images were analyzed by FEI (TALOS F200X, operating at 200 kV) transmission electron microscope (TEM) instruments.

Optical spectroscopy. All measurements on LHP-MSNs in solutions were performed in ethanol using a 10 mm quartz Suprasil cuvette. The optical absorption spectrum was recorded by a Varian Cary 50 spectrometer at normal incidence in dual beam mode, with a spectral resolution of 1 nm. Steady-state PL measurements were performed exciting the samples with a 405 nm pulsed diode laser (Edinburgh Inst. EPL 405, 40 ps pulse width) and collecting with a TM-C10083CA Hamamatsu Mini-Spectrometer. Time-resolved PL was recorded with a Varian Eclipse fluorimeter with time resolution of 12.5 μs collecting for 25 ms exciting with a pulsed lamp at 370 nm with passing band of 2.5 nm.

X-ray Experiments. RL measurements were performed by irradiating the samples at room temperature with a Philips 2274 (steady-state RL spectroscopy) or a Machlett OEG 50 (singlet oxygen production monitoring experiment) X-ray tubes, both with a tungsten target, equipped with a beryllium window and operated at 20 kV and 20 mA. At this voltage, X-rays are generated by the *bremsstrahlung* mechanism. No beam filtering has been applied. RL spectra have been recorded using a homemade apparatus featuring a liquid nitrogen-cooled charge-coupled device (CCD, Jobin-Yvon Symphony II) coupled to a monochromator (Jobin-Yvon Triax 180) with a 100 grooves/mm and a 300 grooves/mm gratings as detection system. The spectra have been corrected for the setup optical response.

Singlet Oxygen Relative Concentration Measurement. The optical probe SOSG has been purchased from Thermo Fisher and used as is. The SOSG powder has been diluted in a 1:10 solution of dimethyl sulfoxide (DMSO) and PBS, which has been used to disperse the $CsPbX_3$-SiO_2 with a concentration of 4 mg/mL. The intensity of the SOSG fluorescence, which is directly proportional to the concentration of singlet oxygen in the environment, has been monitored during the X-ray exposure under continuous-wavelength laser light excitation at 473 nm.

Supplementary Figures

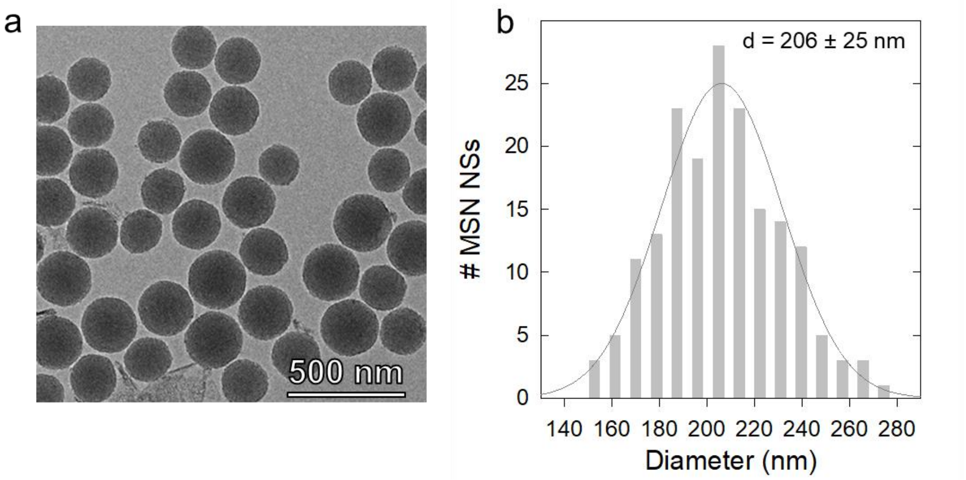

Figure S1. *TEM image (a) and size distribution (b) of pristine SiO_2 NS.*

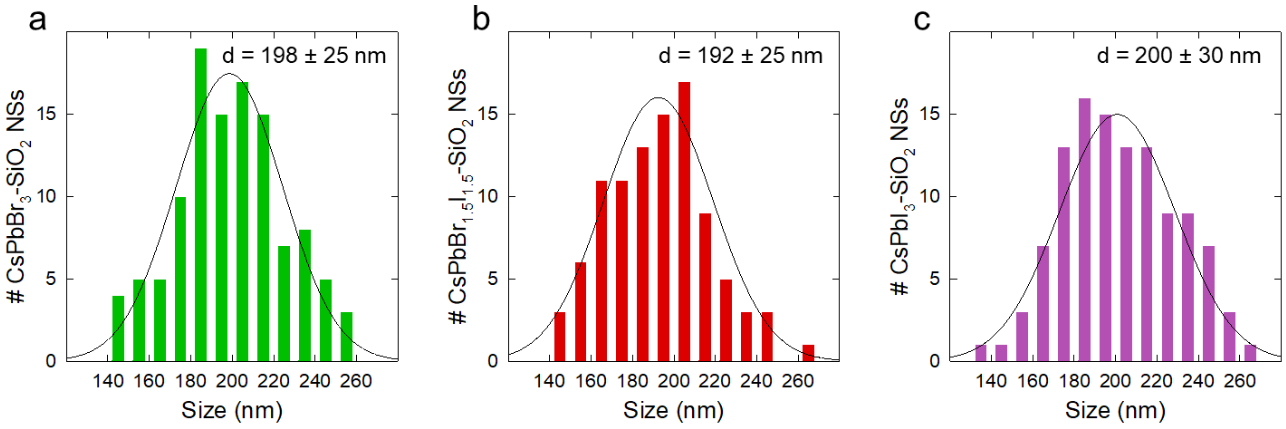

Figure S2. *Size distribution of $CsPbBr_3$-SiO_2 (a), $CsPbBr_{1.5}I_{1.5}$-SiO_2 (b) and $CsPbI_3$-SiO_2 NSs (c).*

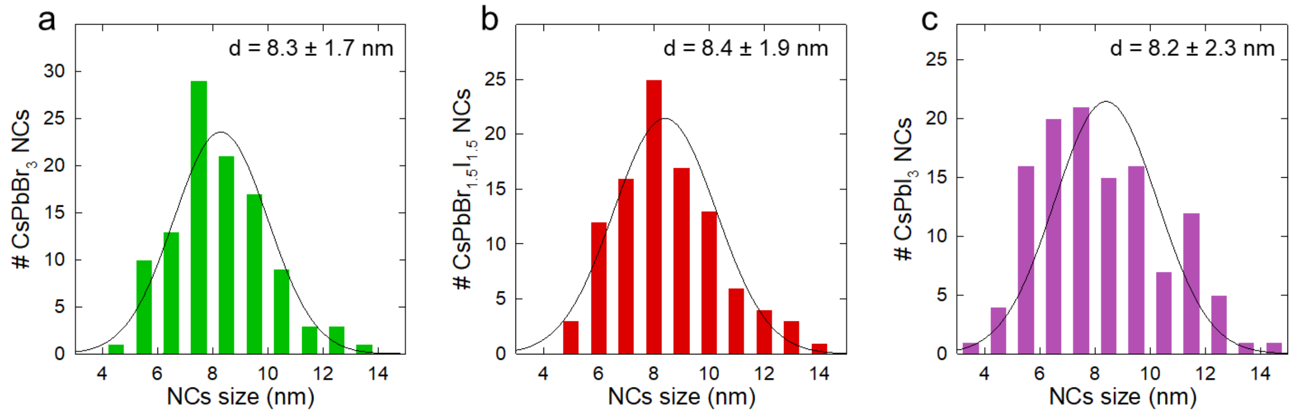

Figure S3. *Size distribution of $CsPbBr_3$ (a), $CsPbBr_{1.5}I_{1.5}$ (b) and $CsPbI_3$ NCs (c) inside SiO_2 NSs*

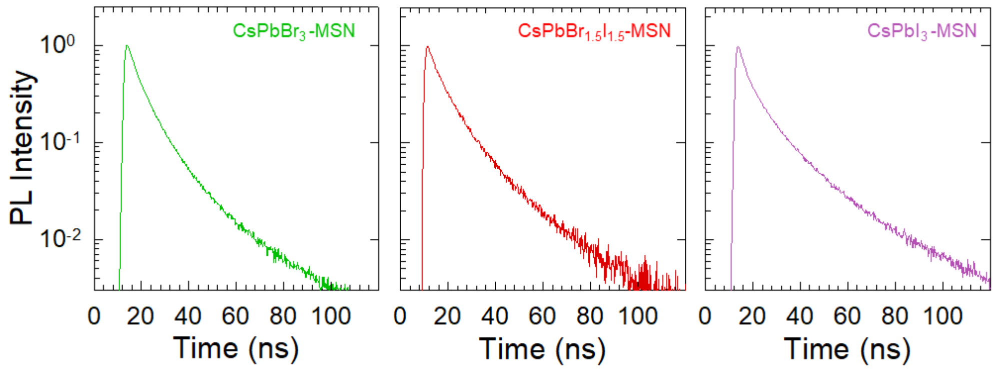

Figure S4. *Time-resolved PL of for $CsPbBr_3$-MSN, $CsPbBr_{1.5}I_{1.5}$-MSN, and $CsPbI_3$-MSN collected at 511 nm, 635 nm and 685 nm, respectively. All LHP-MSN exhibit emission decay dynamics dominated by a fast radiative component with effective exciton lifetime of $<\tau_{CsPbBr3\text{-}MSN}>=5.8$ ns, $<\tau_{CsPbBr1.5I1.5\text{-}MSN}>=6.8$ ns and $<\tau_{CsPbI3\text{-}MSN}>=8.3$ ns.*

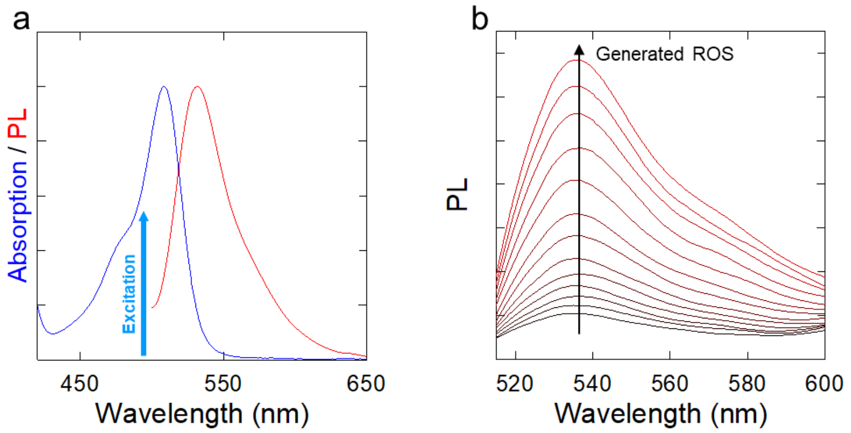

Figure S5. *a) Absorption (blue) and PL emission (red) spectra of SOSG. b) Increase of PL intensity of SOSG excited at 473 nm during X-ray irradiation.*

Evaluation of ROS production rate. A 4 mL PBS solution containing the same concentration of SOSG used in the ROS production experiment (8.3 10^{-5} M) was prepared and 0.5 mg of meso-tetra(4-sulfonatophenyl) porphyrin (H_2TPPS^{4-}), an efficient photo-sensitizer for singlet oxygen generation (yield >60% under UV excitation, structure reported in the inset of **Figure S5**), were added and the final solution and kept under stirring in dark condition until the complete dissolution of H_2TPPS^{4-}. We performed the complete oxidation of SOSG via the photo-sensitizer approach rather than the radio-sensitizer in order to avoid exposing the sample extremely high X-ray dose rate, which may result in undesired sample degradation. The solution was maintained under stirring and exposed to 405 nm while the SOSG PL was monitored using an *in-situ* fiber with 473 nm excitation. UV exposition was maintained until no further increment of SOSG PL was observed, which indicates the complete oxidation of the SOSG in the solution (**Figure S5**). The ratio between the PL collected at the end (corresponding to complete SOSG oxidation) and before UV irradiation (22.3 times higher) was used as to evaluate the fraction of total SOSG moles which are oxidized and thus the ROS moles produced:

$$m_{ROS}(t) = \frac{PL_{SOSG}(t)}{PL_{SOSG}(0)} \frac{1}{22.3} 8.3\ 10^{-5} M * V$$

Where PL_{SOSG} (t) is the PL of the SOSG evaluated after a specific exposure time *t*, 8.3 10^{-5} M is the molarity of the SOSG solution and *V* is the volume of the solution.

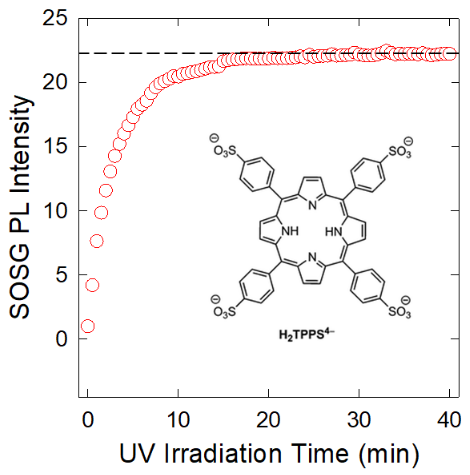

Figure S6. *SOSG PL intensity normalized for its initial value in dispersions containing (H_2TPPS^{4-}) as photosensitizer. In the inset (H_2TPPS^{4-}) structure is reported.*

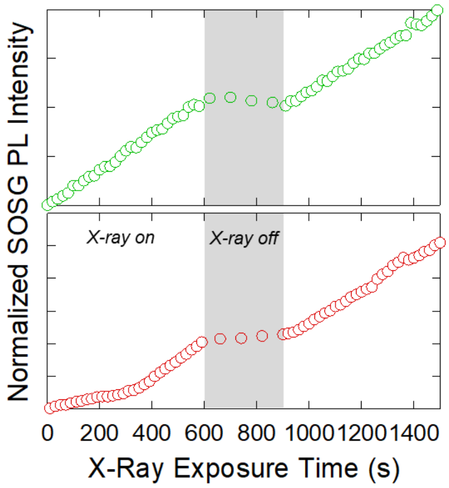

Figure S7. *SOSG PL intensity excited at 473 nm during the full X-ray irradiation sequence in the presence (X-ray ON) and in the absence of simultaneous X-ray irradiation (X-ray OFF) for a solution containing $CsPbBr_3$-SiO_2 NSs (top plot, green circles) and $CsPbBr_{1.5}I_{1.5}$-SiO_2 NSs (bottom plot, red circles).*

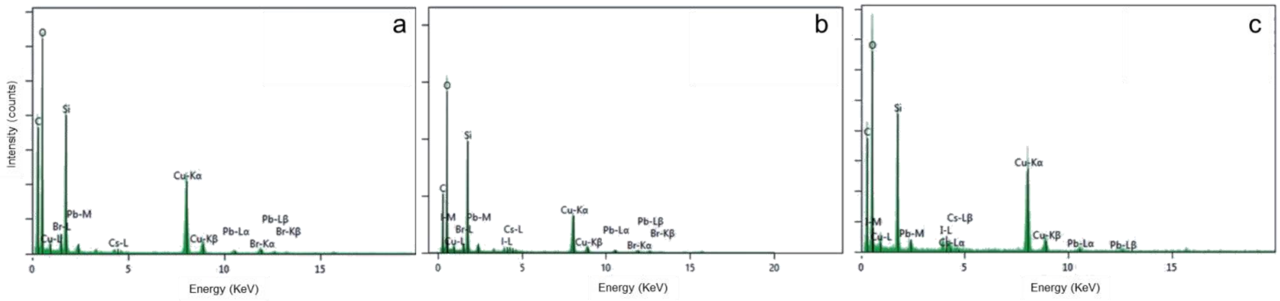

d

	Mass Fraction(%)					
	$CsPbBr_3$		$CsPbBr_{1.5}I_{1.5}$		$CsPbI_3$	
	**Avg**	**SD**	**Avg**	**SD**	**Avg**	**SD**
O	47.08	1.26	45.70	0.69	44.74	0.51
Si	36.79	0.74	36.67	0.55	35.64	0.50
Br	6.92	0.51	3.26	0.18		
I			4.72	0.18	9.47	0.16
Cs	3.47	0.33	3.62	0.12	4.14	0.58
Pb	5.74	0.28	6.03	0.35	6.00	0.12
LHP	16.13	0.98	17.63	0.29	19.85	0.51

	Atomic Fraction(%)					
	$CsPbBr_3$		$CsPbBr_{1.5}I_{1.5}$		$CsPbI_3$	
	**Avg**	**SD**	**Avg**	**SD**	**Avg**	**SD**
O	66.98	1.00	66.48	0.64	66.57	0.46
Si	29.83	0.83	30.39	0.60	30.22	0.45
Br	1.97	0.18	0.95	0.06		
I			0.87	0.03	1.78	0.04
Cs	0.60	0.06	0.63	0.03	0.74	0.10
Pb	0.63	0.04	0.67	0.04	0.69	0.01
LHP	3.20	0.25	3.12	0.05	3.26	0.11

Figure S8. *EDS analysis on $CsPbBr_3$-MSN (a), $CsPbBr_{1.5}I_{1.5}$-MSN (b), and $CsPbI_3$-MSN (c). The corresponding elemental composition extracted from EDS are reported as mass fraction and atomic fraction in table d.*

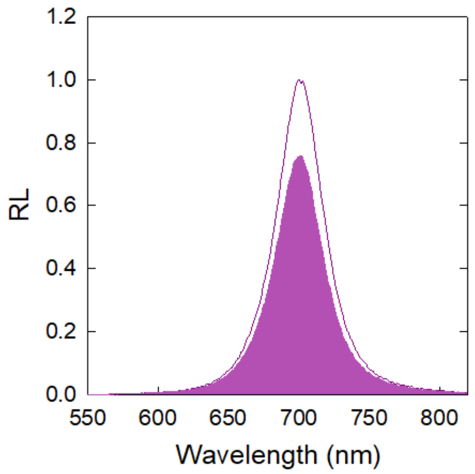

Figure S9. *RL spectra of $CsPbI_3$-MSN before (solid line) and after (shaded area) exposure to X-ray irradiation up to 60 Gy, retaining around 80 % of the initial intensity.*

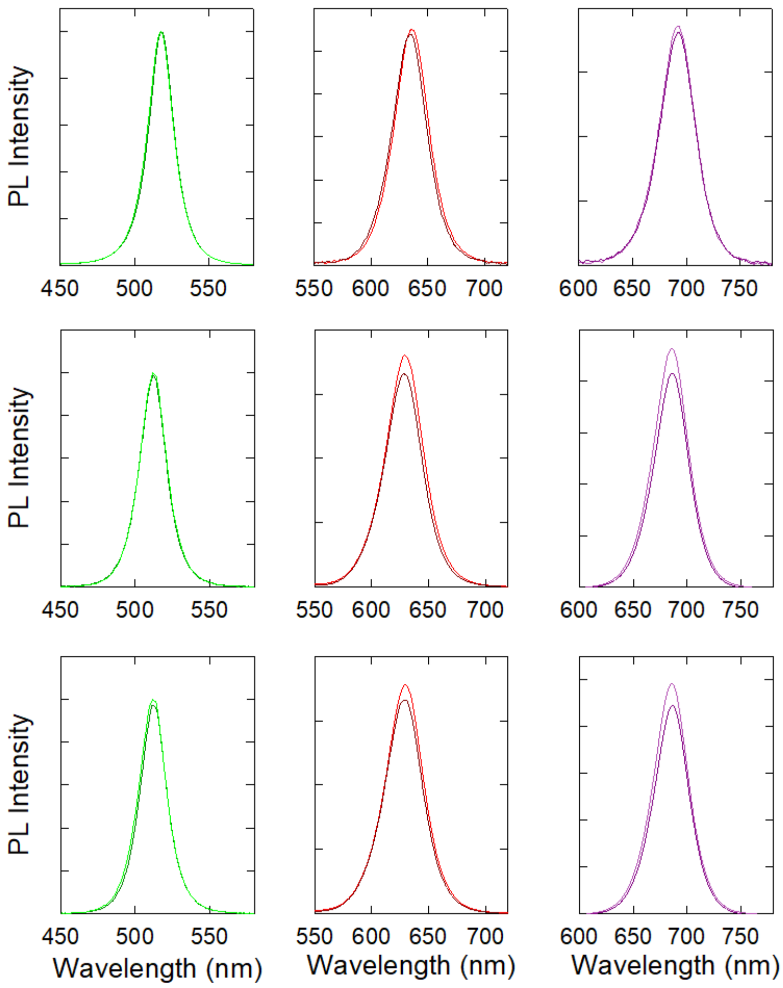

Figure S10. *PL spectra of $CsPbBr_3$-MSN (green lines), $CsPbBr_{1.5}1I_{1.5}$-MSN (red lines) and $CsPbI_3$-MSN (violet lines) collected before (light curves) and after (dark curves) 40 days of storage in different conditions: air (top plots), water (middle plots) and HCl (bottom plots), each showing no significant change in the spectral shape.*
